# Short- and long-term effect of high versus low-to-moderate intensity exercise to optimise health-related quality of life after oncological treatment—results from the Phys-Can project

**DOI:** 10.1007/s00520-022-07016-3

**Published:** 2022-04-07

**Authors:** Anna-Karin Ax, Birgitta Johansson, Johan Lyth, Karin Nordin, Sussanne Börjeson

**Affiliations:** 1 Department of Oncology and Department of Health, Medicine and Caring Sciences, Linköping University, Linköping, Sweden; 2 Department of Immunology, Genetics and Pathology, Uppsala University, Uppsala, Sweden; 3 Department of Public Health and Caring Sciences, Uppsala University, Uppsala, Sweden

**Keywords:** Cancer, Oncological treatment, Exercise, HRQoL

## Abstract

**Purpose:**

This study aimed to evaluate the effect of high intensity (HI) vs low-to-moderate intensity (LMI) exercise on health-related quality of life (HRQoL) up to 18 months after commencement of oncological treatment in patients with breast, colorectal or prostate cancer. In addition, we conducted a comparison with usual care (UC).

**Methods:**

Patients scheduled for (neo)adjuvant oncological treatment (*n* = 577) were randomly assigned to 6 months of combined resistance and endurance training of HI or LMI. A longitudinal descriptive study (UC) included participants (*n* = 89) immediately before the RCT started. HRQoL was assessed by EORTC QLQ-C30 at baseline, 3, 6 and 18 months (1 year after completed exercise intervention) follow-up. Linear mixed models were used to study the groups over time.

**Results:**

Directly after the intervention, HI scored significant (*P* = 0.02), but not clinically relevant, higher pain compared with LMI. No other significant difference in HRQoL was found between the exercise intensities over time. Clinically meaningful improvements in HRQoL over time were detected within both exercise intensities. We found favourable significant differences in HRQoL in both exercise intensities compared with UC over time.

**Conclusion:**

This study adds to the strong evidence of positive effect of exercise and shows that exercise, regardless of intensity, can have beneficial effects on HRQoL during oncological treatment and also for a substantial time after completion of an exercise intervention. In this study, for one year after.

**Implications for cancer survivors:**

Patients can be advised to exercise at either intensity level according to their personal preferences, and still benefit from both short-term and long-term improvements in HRQoL.

**Supplementary Information:**

The online version contains supplementary material available at 10.1007/s00520-022-07016-3.

## Introduction

The incidence of cancer is increasing worldwide, and fortunately more effective oncological treatments are resulting in improved survival [1]. The cancer disease and its treatment often have negative consequences, and compared with the general population, cancer survivors report a lower HRQoL (health-related quality of life) due to adverse outcomes both during treatment and in long-term survivorship [2–6]. A decline in HRQoL negatively affects functioning in daily life and hampers the transition to normal life [4]. Thus, it is important to promote interventions that can reduce or prevent adverse outcomes to enhance the HRQoL for the cancer survivors [7].

There is strong evidence that exercise has short-term beneficial effects on HRQoL and can improve or prevent a decline in physical, emotional, and role functioning and can reduce symptoms while the oncological treatment is ongoing [8–15]. A meta-analysis showed that exercise during, and especially in the months after oncological treatment, had small favourable effects on fatigue and physical functioning compared to usual care up to six-month follow-up [16]; however, long-term effects of exercise on HRQoL are unclear. Combined endurance and resistance training two to three times per week for at least 12 weeks is recommended to improve HRQoL during and after treatment [6, 15]. However, there is insufficient evidence regarding what level of exercise intensity is required to optimise HRQoL most in the short and longer terms. To our knowledge, no study has compared combined endurance and resistance training of high intensity vs. low intensity during oncological treatment or examined the effects on HRQoL up to 1 year after completion of an exercise intervention. A previous RCT compared a supervised exercise programme with combined endurance and resistance training of moderate-to-high intensity, with a home-based walking programme of low intensity. Fewer symptoms of obstipation were found in the moderate-to-high intensity group compared with the low intensity group directly after the exercise intervention; however no differences were found at the 6 months follow-up. [17]. Another RCT compared combined supervised endurance and resistance training of high intensity vs. low intensity after chemotherapy treatment [18, 19]. They found no differences between the different exercise intensities on HRQoL directly after the intervention [18] but did find larger effects of high intensity exercise for social and role functioning compared with low intensity exercise at the one year follow-up after the intervention [19]. Thus, larger randomised studies with a longer follow-up are warranted to further improve the optimal exercise prescription regarding exercise intensity during treatment and to study whether improvements in HRQoL persist over time.

Recently, the short-term main results of the randomised controlled trial Physical Training and Cancer (Phys-Can), determining the effects of a 6-month combined endurance and resistance training programme of (high intensity) HI vs (low-to-moderate intensity) LMI exercise in patients undergoing (neo-)adjuvant treatment, was published [20]. Directly after the intervention was completed, HI exercise yielded statistically significant reduced physical fatigue (the primary outcome) and improved muscle strength and cardiorespiratory fitness compared with LMI exercise. However, there were no other differences between the exercise intensities in overall HRQoL, anxiety, depression, functioning in daily life or sleep. The present study reports the HRQoL in more detail up to 1 year after completion of the exercise intervention in Phys-Can. Hence, this study aimed to evaluate the effect of HI vs LMI exercise on HRQoL up to 18 months after commencement of oncological treatment (12 months after the end of the intervention) in patients with breast, colorectal or prostate cancer. In addition, we conducted a comparison with usual care (UC).

## Patients and method

### Research design and study sample

This is a study on secondary outcomes from the Phys-Can project. The Phys-Can project is a non-blinded RCT (NCT02473003, www.clinicaltrials.gov), with a preceding longitudinal descriptive study with UC to be used as comparison (hereinafter referred as UC). The design of the Phys-Can project is described in detail elsewhere [21]. For the RCT, a 2 × 2 factorial design was used, comparing LMI vs HI exercise with or without additional self-regulatory behaviour change strategies (BCS), for 6 months during oncological treatment. BCS focused on strategies for adherence to the endurance training [21]. However, additional BCS was not beneficial for any of the study outcomes, nor for overall HRQoL directly after the intervention [20]. In the present study, we focused on differences between exercise intensities.

**Fig. 1:**
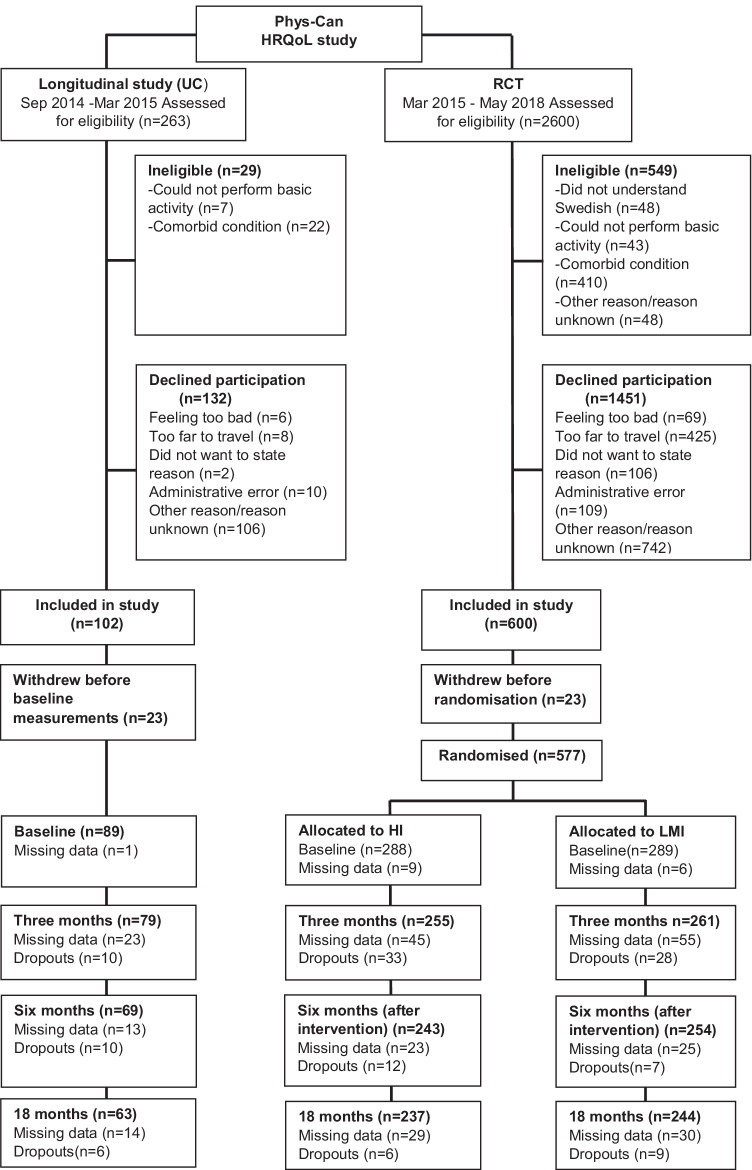
CONSORT diagram of flow of participants through the Phys-Can HRQoL study, including Phys-Can non-randomized study (usual care) and Phys-Can RCT (allocated to HI: High intensity exercise intervention or LMI: Low-to-moderate intensity for six months). Numbers at each time measurement refer to participants remaining in the study. Numbers for missing data refer to the EORTC QLQ C30 questionnaire

Participants were consecutively recruited at three university hospitals in Sweden from September 2014 to March 2015 (UC) and March 2015 to May 2018 (RCT) (Fig. 1). Participants were eligible if diagnosed with breast, colorectal or prostate cancer and scheduled for (neo)adjuvant oncological treatment. Exclusion criteria were inability to perform basic activities of daily living, cognitive disorders, severe emotional instability, or other conditions for which physical exercise is contradicted. The sample size calculation in the RCT was based on the primary outcome of cancer-related fatigue (assessed with the Multidimensional Fatigue Inventory [22]) [21]. The study was approved by the Swedish Ethical Review Authority in Uppsala (Dnr 2014/249). Written informed consent was obtained from all participants.

### Procedure

Baseline measurements were collected before oncological treatment in both the UC and the RCT. Then participants in the RCT were randomly assigned to HI, HI + BCS, LMI, or LMI + BCS (Fig. 1). The randomisation was computer-generated with a random allocation sequence (1:1:1:1) and carried out with eight patients per block within each stratum (three hospitals and three diagnoses).

### Intervention

The exercise programme consisted of endurance training and resistance training for 6 months and was initiated when the oncological treatment started, as described in detail elsewhere [20, 21]. For endurance training (home-based), participants in HI performed interval sessions twice a week, alternating between 2 min of exercise (e.g. running) at an exercise intensity of 80–90% of the heart rate reserve (HRR) with 2 min of active rest (e.g. walking). Participants started with 5 intervals and were adding intervals up to a maximum of 10 intervals. In LMI, participants performed at least 150 min per week (i.e. walking and bicycling) at an exercise intensity of 40–50% of the HRR in bouts of at least 10 min. HRR was determined from VO2max test performed at baseline. Heart rate monitors were used for monitoring of exercise intensity. The resistance training was supervised and performed twice a week in groups in a public gym. The HI group performed 6 repetitions maximum (RM) × 3 sets (first weekly session) and 10 RM × 3 sets (second weekly session). The LMI group performed 12 repetitions (50% of 6 RM) × 3 sets (first weekly session) and 10 repetitions (50% of 10 RM) × 3 sets (second weekly session). The progression was based on testing of 6 and 10 RM every 4–6 weeks. The resistance training was performed on machines: seated leg press, chest press, leg extension, seated row, seated leg curl and seated overhead press using dumbbells. In addition, core exercises were performed. Participants were closely monitored. The coaches checked for intensity and overall adherence to the exercise protocol and gave feedback on the exercise being performed. Adverse events during exercise were assessed by both coaches and participants and revealed mainly minor musculoskeletal injuries and/or discomfort [23].

### Timing of assessments and study measures

Follow-up data collection was completed in November 2019. Participants completed the HRQoL questionnaire at baseline, after 3 months (mid-intervention for the RCT), after 6 months (end of the intervention for the RCT) and after 18 months (1 year after the end of the intervention for the RCT). Medical background data were collected from the medical records and the Swedish National Quality Registers. Socio-demographic data were self-reported (study-specific questions). HRQoL was assessed by The European Organisation for Research and Treatment of Cancer, Quality of life Questionnaire C30 (EORTC QLQ-C30) [24, 25], which is a questionnaire validated for the cancer population, and consists of a 30-item questionnaire, covering a global health status/quality of life (QoL) scale, five functioning scales, three symptom scales and six items concerning symptoms. All scales and single-item measures were transformed to scores in the range 0–100. A higher score on the global status scale and the functional scales denotes a high level of health and functioning, while a higher score on the symptomatic scale denotes a high level of symptom burden [26]. In addition, participants with breast cancer completed EORTC QLQ-BR23 [27], participants with colorectal cancer completed EORTC QLQ-CR29 [28] and participants with prostate cancer completed EORTC QLQ-PR25 [29].

### Statistical analysis

**Fig. 2:**
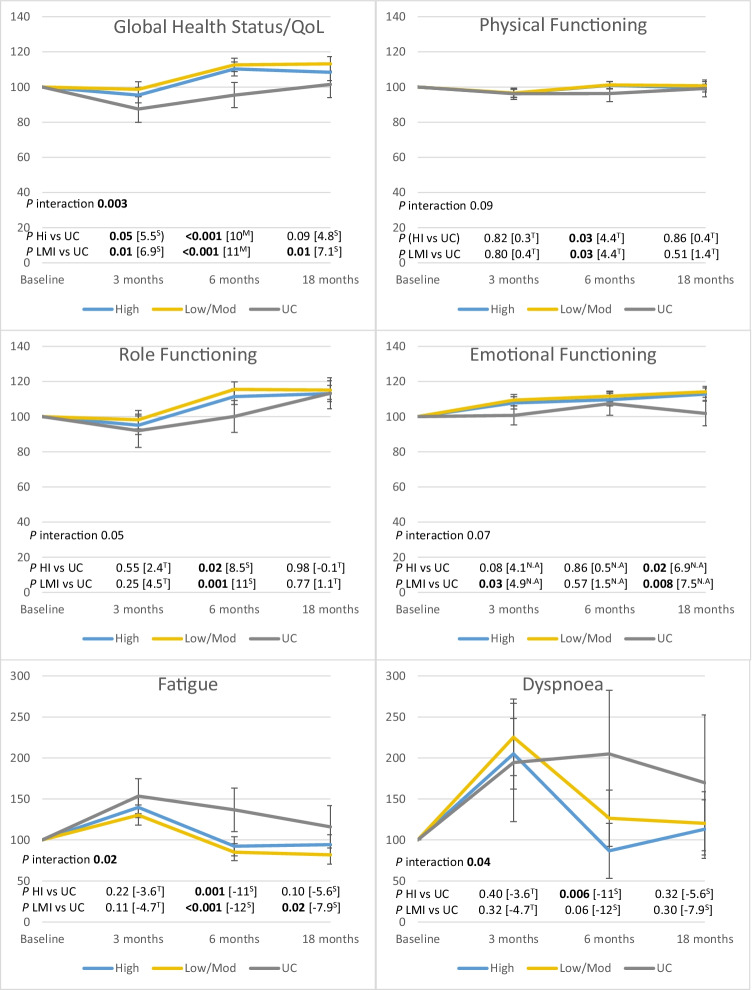
Significant p-values of functioning and symptoms of EORTC QLQ C30 for high intensity (HI), low-to-moderate intensity (LMI) and usual care (UC) over time. Note: Baseline measurements were scaled to 100, and changes are presented in percentages. A high score for the global health status and functional scale represents a high QoL and a high level of functioning. A high score for the symptom scale/item represents a high level of symptoms/problems. Unscaled observed mean differences between groups are presented within the brackets. Clinically relevant differences were defined as T = trivial (unlikely to have a clinically relevance), S = small (subtle but nevertheless clinically relevant), M = medium (likely to be clinically relevant but to a lesser extent) and N/A = No guidelines applicable, by Cocks et al. 2010

Analysis was conducted as intention-to-treat and carried out using IBM statistics SPSS 25. Descriptive analysis was used to present background characteristics and the scores of the HRQoL outcome. To compare background characteristics between the groups, a Chi2-test was used for categorical data and ANOVA for continuous data. Linear mixed models were used to estimate the longitudinal changes of each HRQoL variable. A normal distribution assumption was made for all HRQoL variables used in the linear mixed models, but robust covariances were used to allow violations of the model assumption. Time was considered categorical, and in all models, an interaction term between time and group was included. The baseline measurement of each outcome and age, education, hospital, cancer diagnosis and chemotherapy (Yes/No) were included as potential confounders or auxiliary variables. Estimated marginal means were calculated from the models, and contrasts were used to calculate all adjusted pairwise p values between groups for each measurement. We did not correct for multiplicity, given the exploratory nature of the study. To illustrate the percentage change between groups (Fig. 2), estimated marginal means and corresponding 95% confidence intervals (CI) were rescaled by dividing all values in each group by the baseline value for that group. *P* values < 0.05 were considered statistically significant. To estimate clinically meaningful changes over time, we used guidelines with thresholds for deterioration/improvements in points of each scale of HRQoL, as trivial, small, medium or large [30]. To estimate clinically relevant differences between groups, we used guidelines with thresholds of mean difference in points of each scale of HRQoL, as trivial, small, medium or large [31]. Logistic regression analysis was conducted to identify possible factors associated with dropouts (age, diagnosis, hospital, treatment, educational level, life situation and comorbidities).

## Results

Overall, 577 participants were randomly assigned to HI (*n* = 288) and LMI (*n* = 289) in the RCT, and 89 participants were included in the UC study. Socio-demographic and medical background characteristics were well balanced across groups, except that a lower proportion of participants with breast cancer received chemotherapy in UC (*P* < 0.01) compared with the RCT. The mean age was 58 years (22–85 years); 539 (81%) were female; 530 (80%) had breast cancer; 109 (16%) had prostate cancer and 27 (4%) had colorectal cancer (Table 1). Baseline questionnaires were completed for 97% (HI), 98% (LMI) and 99% (UC). The response rate was > 71% for all measurements. Participants in the UC study were more likely to drop out compared with the RCT, HI (OR:0.46, 95% CI, 0.27–0.81, *P* < 0.01) and LMI: (OR:0.32, 95% CI, 0.18–0.57, *P* < 0.01). From baseline to 18 months, there was a 28% drop-out from UC, 18% from HI and 16% from LMI (Fig. 1). There were no systematic non-answers to any HRQoL item.

**Table 1:** Medical background characteristics of high- and low-to-moderate intensity exercise and usual care in patients undergoing (neo)adjuvant oncological treatment

	HI (*n* = 288)	LMI (*n* = 289)	UC (*n* = 89)	*P* (between groups)
Age (years), mean (SD)	58 (12)	58 (12)	58 (11)	0.997^1^
Sex, n (%)				0.827^2^
Male	57 (20)	55 (19)	15 (17)	
Female	231 (80)	234 (81)	74 (83)	
Number of comorbidities				0.624^2^
0	113 (42)	109 (39)	37 (45)	
1	84 (31)	88 (31)	29 (35)	
2	46 (17)	58 (21)	11 (13)	
3 +	27 (10)	24 (8.6)	5 (6.1)	
Breast cancer	228	229	73	
Stage				0.301^2,3^
I (T1N0M0) and in situ	122 (61)	113 (56)	39 (66)	
II (T1-2N1M0, T2-3N0M0)	74 (37)	79 (39)	20 (34)	
III (T1-2N2M0, T3N1-2M0)	5 (2.5)	11 (5.4)	0 (0)	
Chemotherapy/target therapy total	118 (65)	127 (66)	24 (41)	0.002^2^
Anthracycline and/or Taxane	84 (71)	91 (78)	19 (79)	
Anthracycline and/or Taxane + target therapy + antibody	34 (29)	36 (29)	5 (21)	
Radiotherapy	170 (81)	177 (84)	44 (75)	0.261^2^
Endocrine therapy	147 (70)	164 (77)	48 (79)	0.135^2^
Prostate cancer	49	48	12	
TNM classification				
T1a-cN0M0	23 (50)	18 (41)	7 (64)	0.649^2^
T2N0M0	16 (35)	17 (39)	2 (18)	
T3-4N0M0, T1c-4N1M0	7 (15)	9 (21)	2 (18)	
Radio- and/or brachy therapy	44 (100)	45 (100)	11 (100)	-
Endocrine therapy	25 (57)	25 (53)	7 (64)	0.810^2^
Colorectal cancer	11	12	4	
Stage				^4^
II (T3-4N0M0)	2 (20)	3 (27)	1 (25)	
III- IV (T2-4N1-2M0, T3N1M1)	8 (80)	8 (73)	3 (75)	
Chemotherapy				
Oxaliplatin and/or Capecitabine	9 (100)	11 (100)	4 (100)	^4^

HI = high intensity exercise; LMI = low-to-moderate intensity exercise, UC = usual care. TNM classification according to AJCC,8 2017. T: tumour size. N: lymph node status.1. One-way ANOVA analysis, Bonferroni post-hoc test. 2. Chi-2-test. 3. Stage II and III were merged in the analysis. 4. Too few numbers to perform analysis

### HRQoL

#### High intensity and low-to-moderate intensity

*At 6 months*, we found statistically significant higher pain in participants randomised to HI exercise compared to LMI exercise; however, this difference was not clinically relevant (Table 2). The most prominent clinically meaningful changes over time within both exercise intensities were improved global health status/QoL, reduced symptoms of appetite loss, and improved social, emotional, and role functioning (Table 2). When exploring each group over time, from the time period when the intervention was completed until 1 year after, significant changes within each group were only found for cognitive functioning (HI: 95% CI, − 4.9 to − 0.3 and LMI: 95% CI − 4.9 to − 0.5), which improved in both exercise intensities. This indicated that the exercise-induced improvements at the end of the intervention persisted in the longer term.

**Table 2:** Longitudinal crude mean scores and estimated clinically relevant differences between groups and meaningful changes within groups of EORTC QLQ- C30, of high- and low-to-moderate intensity exercise and usual care

*Mean (SD)*		Baseline	3 months	6 months	18 months	Between groups differences at 6 months (95% CI and *P*)		Clinically relevant differences between groups at 6 months^a^	Clinically meaningful changes within group from baseline to 6 months^b^	Between groups differences at 18 months (95% CI and *P*)		Clinically relevant differences between groups at 18 months^a^	Clinically meaningful changes within group from baseline to 18 months^b^
Maximum number of respondents	HI	279	210	220	208								
	LMI	283	206	229	214								
	UC	88	56	56	49								
Global health status	HI	67 (20)	64 (23)	75 (19)	74 (19)				+ 7 Small*				+ 6 Small*
	LMI	65 (20)	65 (22)	74 (19)	74 (18)				+ 9 Medium*				+ 9 Medium*
	UC	69 (19)	64 (20)	67 (21)	74 (18)				-3 Trivial				+ 1 Trivial
	HI vs LMI					− 0.6 (− 3.6 to 2.4)	0.70	-1 Trivial		-2.3 (-5.7 to 1.1)	0.18	-2 Trivial	
	HI vs UC					10 (5.1–16)	< 0.01	+ 10 Medium*		4.8 (-0.8 to 10)	0.09	** + 5 Small**	
	LMI vs UC					10 (5.8–16)	< 0.01	+ 11 Medium*		7.1 (1.7 to 13)	0.01	+ 7 Small*	
Functioning													
Physical functioning	HI	89 (13)	85 (16)	90 (13)	80 (14)				+ 1 Trivial				-1 Trivial
	LMI	88 (14)	85 (15)	90 (12)	89 (14)				+ 1 Trivial				+ 1 Trivial
	UC	90 (11)	88 (11)	88 (14)	90 (13)				-3 Trivial				0 Trivial
	HI vs LMI					0 (-2.0 to 2.0)	0.99	0 Trivial		-1.0 (-3.4 to 1.3)	0.38	+ 1 Trivial	
	HI vs UC					4.4 (0.5 to 8.3)	0.03	+ 4 Trivial*		0.4 (-3.9 to 4.7)	0.86	0 Trivial	
	LMI vs UC					4.4 (0.5 to 8.3)	0.03	+ 4 Trivial*		1.4 (-2.9 to 5.7)	0.51	+ 1 Trivial	
Role functioning	HI	74 (23)	70 (32)	83 (24)	84 (25)				+ 9 Small*				+ 10 Small*
	LMI	73 (29)	72 (30)	86 (21)	85 (23)				+ 12 Small*				+ 11 Small*
	UC	77 (27)	73 (30)	80 (24)	90 (19)				0 Trivial				+ 10 Small*
	HI vs LMI					-2.8 (-6.7 to 1.3)	0.18	-3 Trivial		-1.2 (-5.9 to 3.5)	0.62	-1 Trivial	
	HI vs UC					8.5 (1.5 to 16)	0.02	+ 9 Small*		-0.1 (-7.1 to 6.9)	0.98	0 Trivial	
	LMI vs UC					11 (4.4 to 18)	< 0.01	0 Trivial*		1.2 (-6.1 to 8.2)	0.77	+ 1 Trivial	
Emotional functioning	HI	72 (22)	77 (21)	79 (22)	81 (20)				+ 7 Small*				+ 9 Small*
	LMI	71 (21)	78 (21)	80 (19)	82 (17)				+ 8 Small*				+ 10 Medium*
	UC	75 (21)	77 (23)	82 (20)	79 (19)				+ 5 Trivial*				+ 1 Trivial
	HI vs LMI					-1.0 (-3.9 to 1.9)	0.50	-1 NA		-0.5 (-3.6 to 2.6)	0.74	-1 NA	
	HI vs UC					0.5 (-4.9 to 5.9)	0.86	+ 1 NA		6.9 (1.2 to 12.6)	0.02	+ 7 NA	
	LMI vs UC					1.5 (-3.7 to 6.7)	0.57	+ 1 NA		7.5 (1.9 to 13.0)	0.01	+ 7 NA	
Cognitive functioning	HI	82.4 (20.4)	78 (23)	83 (21)	85 (18)				+ 2 Trivial				+ 2 Trivial
	LMI	82 (21)	78 (23)	81 (19)	84 (17)				-1 Trivial				+ 2 Trivial
	UC	84 (21)	83 (20)	83 (19)	86 (18)				-1 Trivial				-1 Trivial
	HI vs LMI					0.6 (-2.4 to 3.7)	0.68	+ 1 Trivial		0.6 (-2.5 to 3.7)	0.72	+ 1 Trivial	
	HI vs UC					1.2 (-4.8 to 7.2)	0.70	+ 1 Trivial		3.1 (-1.5 to 7.7)	0.19	+ 3 Trivial	
	LMI vs UC					0.5 (5.4 to 6.5)	0.86	+ 1 Trivial		2.5 (-2.0 to 7.1)	0.28	+ 3 Trivial	
Social functioning	HI	81 (22)	75 (25)	83 (21)	85 (22)				+ 2 Trivial				+ 3 Trivial*
	LMI	78 (23)	73 (25)	84 (20)	85 (20)				+ 5 Small*				+ 6 Small*
	UC	83 (21)	77 (25)	82 (24)	87 (20)				-1 Trivial				+ 3 Trivial
	HI vs LMI					-2.3 (-5.7 to 1.0)	0.17	-2 Trivial		-2.2 (-6.1 to 1.6)	0.26	-2 Trivial	
	HI vs UC					3.4 (-3.3 to 10)	0.32	3 Trivial		1.6 (-5.2 to 8.3)	0.65	2 Trivial	
	LMI vs UC					5.7 (-1.0 to 12)	0.09	+ 6 **Small**		3.8 (-3.0 to 11)	0.27	+ 4 Trivial	
Symptoms													
Fatigue	HI	27 (20)	38 (25)	25 (20)	25 (21)				-2 Trivial				-1 Trivial
	LMI	30 (23)	37 (25)	25 (19)	25 (19)				-4 Trivial*				-5 Small*
	UC	23 (17)	34 (23)	31 (24)	26 (22)				+ 9 Small*				+ 4 Trivial
	HI vs LMI					1.0 (-2.3 to 4.2)	0.56	+ 1 Trivial		2.3 (-1.2 to 5.7)	0.20	+ 2 Trivial	
	HI vs UC					-11 (-18 to -4.5)	< 0.01	-11 Small*		-5.6 (-12 to 1.1)	0.10	-6 **Small**	
	LMI vs UC					-12 (-19 to -5.6)	< 0.01	-12 Small*		-7.9 (-14 to -1.2)	0.02	-8 Small*	
Nausea and vomiting	HI	3.5 (9.3)	7.1 (13)	2.9 (9.2)	1.9 (6.3)				-1 Trivial				-2 Trivial*
	LMI	3.1 (8.1)	6.5 (12)	2.3 (7.8)	2.5 (8.3)				-1 Trivial				0 Trivial
	UC	3.0 (7.4)	6.5 (13)	1.8 (7.6)	4.1 (18)				-1 Trivial				1 Trivial
	HI vs LMI					0.3 (-1.3 to 1.9)	0.74	0 Trivial		-1.0 (-2.6 to 0.6)	0.21	-1 Trivial	
	HI vs UC					1.3 (-0.9 to 3.4)	0.26	+ 1 Trivial		-3.0 (-7.7 to 1.8)	0.22	-3 Trivial	
	LMI vs UC					1.0 (-1.1 to 3.1)	0.36	-2 Trivial		-1.9 (-6.7 to 2.8)	0.42	+ 1 Trivial	
Pain	HI	19 (22)	24 (27)	20 (24)	21 (24)				+ 1 Trivial				+ 3 Trivial
	LMI	22 (24)	23 (26)	17 (21)	19 (23)				-5 Small*				-2 Trivial
	UC	17 (19)	17 (23)	17 (22)	19 (25)				+ 2 Trivial				+ 4 Trivial
	HI vs LMI					4.7 (0.9 to 8.5)	0.02	+ 5 Trivial*		3.8 (-0.6 to 8.2)	0.09	+ 4 Trivial	
	HI vs UC					-0.2 (-6.7 to 6.3)	0.95	0 Trivial		-1.3 (-8.9 to 6.4)	0.74	-1 Trivial	
	LMI vs UC					-4.9 (-11 to 1.6)	0.14	-5 Trivial		-5.1 (-13 to 2.6)	0.19	-5 Trivial	
Dyspnoea	HI	10 (19)	20 (28)	8.5 (19)	10 (20)				-1 Trivial				+ 1 Trivial
	LMI	8.8 (17)	19 (26)	9.8 (18)	9.4 (20)				+ 2 Trivial*				+ 2 Trivial
	UC	4.9 (14)	12 (22)	13 (24)	11 (24)				+ 9 Small*				6 **Small**
	HI vs LMI					-3.0 (-6.4 to 0.4)	0.08	-3 Trivial		0.1 (-3.7 to 3.8)	0.97	0 Trivial	
	HI vs UC					-9.2 (-16 to -2.7)	0.01	-9 Small*		-3.7 (-11 to 3.5)	0.32	-4 **Small**	
	LMI vs UC					-6.2 (-13 to 0.3)	0.06	-6 **Small**		-3.8 (-11 to 3.4)	0.30	-4 **Small**	
Insomnia	HI	30 (30)	36 (30)	31 (30)	28 (29)				+ 2 Trivial				-1 Trivial
	LMI	31 (30)	32 (32)	30 (30)	27 (28)				0 Trivial				-2 Trivial
	UC	29 (30)	33 (31)	31 (31)	31 (31)				+ 2 Trivial				+ 5 Trivial
	HI vs LMI					1.4 (-3.5 to 6.3)	0.58	+ 1 Trivial		1.3 (-3.8 to 6.4)	0.61	+ 1 Trivial	
	HI vs UC					-0.1 (-8.6 to 8.4)	0.98	0 Trivial		-5.2 (-15 to 4.2)	0.28	-5 **Small**	
	LMI vs UC					-1.5 (-9.9 to 6.9)	0.73	-1 Trivial		-6.5 (-16 to 3.0)	0.18	-6 **Small**	
Appetite loss	HI	9.5 (20)	12 (22)	4.7 (14)	6.3 (18)				-5 Small*				-3 Small*
	LMI	10 (19)	13 (24)	4.3 (12)	4.7 (16)				-5 Small*				-5 Small*
	UC	6.4 (19)	9.5 (20)	4.8 (17)	6.1 (22)				-2 Trivial				+ 1 Trivial
	HI vs LMI					0.3 (-2.2 to 2.9)	0.79	0 Trivial		1.7 (-1.7 to 5.1)	0.33	2 Trivial	
	HI vs UC					-2.3 (-6.9 to 2.4)	0.34	-2 Trivial		-3.9 (-10 to 2.2)	0.21	-4 Trivial	
	LMI vs UC					-2.6 (-7.1 to 1.9)	0.26	-3 Trivial		-5.6 (-12 to 0.5)	0.07	-6 **Small**	
Constipation	HI	5.5 (15)	13 (23)	6.4 (16)	6.6 (16)				+ 1 Trivial				+ 1 Trivial
	LMI	6.8 (17)	13 (22)	9.3 (20)	8.9 (19)				+ 2 Trivial				+ 2 Trivial
	UC	6.4 (16)	9.5 (23)	7.7 (18)	10 (23)				+ 2 Trivial				+ 6 **Small**
	HI vs LMI					-1.8 (-4.8 to 1.3)	0.26	-2 Trivial		-0.6 (-3.8 to 2.5)	0.69	-1 Trivial	
	HI vs UC					-2.1 (-6.6 to 2.4)	0.35	-2 Trivial		-5.0 (-12 to 1.8)	0.15	-5 Trivial	
	LMI vs UC					-0.4 (-4.9 to 4.1)	0.86	0 Trivial		-4.4 (-11 to 2.4)	0.20	-4 Trivial	
Diarrhoea	HI	8.4 (19)	14 (23)	7.1 (18)	7.0 (18)				-1 Trivial				-1 Trivial
	LMI	8.4 (19)	15 (24)	9.2 (19)	7.5 (19)				1 Trivial				-1 Trivial
	UC	7.6 (19)	14 (21)	11 (22)	8.8 (22)				+ 3 Trivial				+ 1 Trivial
	HI vs LMI					-1.9 (-5.4 to 1.6)	0.28	-2 Trivial		-0.6 (-4.0 to 2.7)	0.71	-1 Trivial	
	HI vs UC					-4.2 (-9.7 to 1.3)	0.13	-2 Trivial		-5.2 (-12 to 1.5)	0.13	-5 Trivial	
	LMI vs UC					-2.3 (-7.8 to 3.3)	0.42	0 Trivial		-4.5 (-11 to 2.1)	0.18	-4 Trivial	
Financial difficulties	HI	9.8 (21)	9.9 (21)	9.4 (22)	7.4 (18)				-1 Trivial				-1 Trivial
	LMI	12.1 (24)	11 (23)	9.8 (19)	5.9 (16)				-2 Trivial				-5 Small*
	UC	9.8 (21)	8.5 (21)	7.9 (22)	3.4 (12)				-0 Trivial				-4 **Small**
	HI vs LMI					0.8 (-2.1 to 3.7)	0.58	+ 1 Trivial		3.1 (-0.3 to 6.6)	0.07	+ 3 Trivial	
	HI vs UC					-1.2 (-6.4 to 4.2)	0.68	-1 Trivial		2.6 (-1.1 to 6.2)	0.16	+ 3 Trivial	
	LMI vs UC					-1.9 (-7.2 to 3.3)	0.47	-2 Trivial		-0.6 (-4.1 to 3.0)	0.75	-1 Trivial	

Results of EORTC QLQ-C30**,** European Organisation for Research and Treatment of Cancer Quality of Life Questionnaire Core 30; are presented as means ± S.D. (range). A high score for the global health status/QoL and functional scale represents a high level of the global health status/QoL and functioning. A high score for a symptom scale/item represents a high level of symptoms/problems. a: Interpreted as clinically relevant as defined by Cocks et al. 2011 b: Interpreted as clinically meaningful as defined by Cocks et al. 2012: Trivial = no/unlikely difference, S = small change of subtle clinical relevance, M = medium change of likely clinical relevance, NA = no available guidelines. Differences are adjusted according to baseline measurement of each outcome, and age, education, hospital, cancer diagnosis and treatment. * = indicates significant differences. Bold font indicates clinically meaningful. HI = high intensity exercise; LMI = low-to-moderate intensity exercise, UC = usual care

#### High intensity, low-to-moderate intensity and usual care

Additionally, we compared the exercise intensities with UC and found statistically significant differences in favour of the exercise groups compared with UC. *At 3 months*, both the HI and LMI groups reported better global health status/QoL (mean difference = 5.5; 95% CI, 0–11 and mean difference = 6.9; 95% CI, 1.5–12, respectively), and the LMI group reported better emotional functioning (mean difference = 4.9; 95% CI, 0.4–9.4). *At 6 months*, both the HI and LMI groups reported better global health status/QoL, physical- and role functioning. Both the HI and LMI groups reported less fatigue, and the HI group reported less dyspnoea (Table 2). *At 18 months*, both the HI and LMI groups reported better emotional functioning. The LMI group reported better global health status/QoL and less fatigue (Table 2). The significant changes over time are presented in Fig. 2.

### Diagnosis-specific HRQoL

#### High intensity and low-to-moderate intensity

No statistically significant differences were observed between the exercise intensities over time.

#### High intensity, low-to-moderate intensity, and usual care

Statistically significant differences were observed at the 3-month measurement between the exercise intensities and UC. In participants with breast cancer, both the HI and LMI groups scored worse for sexual functioning (mean difference =  − 5.5; 95% CI, − 10 to 1.1 and mean difference =  − 6.3; 95% CI, − 11 to − 1.9), and there was more hair loss in the LMI group (mean difference = 8.7; 95% CI, 0.2–17) compared with UC. In participants with prostate cancer, both the HI and LMI groups reported better sexual functioning (mean difference = 21.8; 95% CI, 5.4 to 38 and mean difference = 25.6; 95% CI, 9.7–42) and the HI group reported more bowel symptoms (mean difference = 5.8; 95% CI, 1.0–11) compared with UC. The sample of participants with colorectal cancer was too small to perform any diagnosis-specific analysis (Supplement material).

## Discussion

This large study demonstrates novel and clinically important results on HRQoL, directly comparing HI vs LMI exercise for 6 months during oncological treatment and up to 1 year after the intervention was completed. We found no significant differences on HRQoL between the exercise intensities over time, except for participants randomised to HI exercise who reported significant higher pain compared with LMI exercise directly after the intervention was completed. However, this difference was not clinically relevant. Small to medium beneficial clinical changes within both exercise intensities were persistent up to 1 year after the intervention was completed. Thus, our findings indicate that to improve or prevent a decline in HRQoL, combining resistance and endurance training of either LMI or HI is recommended. In addition, the present study confirmed that supervised exercise during treatment could be beneficial for many aspects of HRQoL compared with UC (although not randomised, discussed in detail below) and also up to 18 months after commencement of treatment.

Our results show that exercise, irrespective of intensity level, could have a beneficial impact on HRQoL up to 1 year after the end of the exercise intervention. This confirms and adds to the findings of a meta-analysis covering 66 studies with different methodologies and cancer patient cohorts, showing a beneficial effect but only immediately after the end of interventions [14]. In addition, our study showed that improvements persisted, regardless of the exercise intensity, up to 1 year after the intervention. Due to methodological differences in the included studies, the meta-analysis could only be conducted for overall QoL, global health status/QoL and physical function, whereas in our study, we were able to investigate all aspects of HRQoL.

In the shorter term, directly after the intervention was completed, the only significant, but not clinically relevant difference between the exercise intensity groups in our study, was higher symptoms of pain with HI exercise compared to LMI. Since other studies have shown that high intensity exercise is beneficial in reducing pain compared to usual care [17, 32], it remains unclear to what degree the higher pain in our study is attributed to HI exercise. Thus, more research is needed to explore this finding. Our results of no significant differences between the exercise intensities of other HRQoL outcomes confirm a smaller randomised study by van Waart et al. [17]. However, van Waart et al. did not directly compare exercise intensities of an exercise programme comprising both endurance and resistance training as we did. Another exercise trial by Kampshoff et al. also reported no significant differences on HRQoL between HI and LMI exercise directly after the exercise intervention [18]. However, Kampshoff et al. conducted an exercise intervention after completion of chemotherapy, and it is proven to be more beneficial to start exercise during chemotherapy.

In the longer term, 1 year after completion of the exercise intervention, no significant difference between the exercise intensities was found in our study, confirming the results of van Waart et al. [17]. However, they only followed up for 6 months after completion of the exercise interventions. In contrast to our results, Kampshoff et al. found larger effects of high intensity exercise on HRQoL compared to low-to-moderate exercise [19].

Our study also reports novel information about clinically meaningful changes of HRQoL within both the HI and LMI groups that persisted 1 year after the intervention. These are important results for cancer survivors, since they are at risk of developing long-term symptoms [6]. Our results are surprisingly positive, as we would expect these outcomes to further deteriorate when the intervention has ended. The intervention in our study lasted for as long as 6 months, which is an appropriate period of time to establish physically active behaviour [33]. A majority of the participants maintained physical active 1 year after the intervention ended [34]. Thus, a reasonable explanation for the maintained improvements might be that the exercise intervention introduced participants to the habit of exercise, which they continued with. The adherence to the exercise programme in Phys-Can RCT had an acceptable rate of ≥ 50% [35]; however, we did not control for whether the participants continued to perform the exercise programme on their own.

Our results confirm previous findings of exercise trials [8, 36], with short-term effects of improved global health status/QoL [14], functioning [14, 17, 32] and reduced fatigue [17, 32] from exercise during oncological treatment compared to usual care. These findings strengthen the hypothesis that supervision in groups can improve aspects of HRQoL [14, 37, 38]. Thus, this study provides important confirmatory findings to support the exercise recommendations [15]. Improvements in HRQoL can be expected when combining supervised resistance and endurance training for a longer period of time.

Our results also confirm that exercise during treatment can have beneficial effects on HRQoL compared to usual care in the longer term. Similar results regarding better emotional functioning and reduced symptoms of fatigue were found by Mijwel et al. at the 12-month follow-up after commencement of treatment of a programme of high intensity aerobic interval training combined with moderate intensity aerobic training [39]. However, comparison with other studies is difficult due to differences in intervention characteristics and follow-up times. Thus, more studies are needed to confirm the long-term effects of exercise.

Participants with breast cancer reported more hair loss and lower sexual functioning with both exercise intensities compared with UC. It is unlikely that these results are due to the exercise. The UC group received less chemotherapy than those in the RCT, and this could explain the lower mean values of side effects of the chemotherapy [40].

The strengths of our study were a long-term follow-up 1 year after completion of the exercise intervention and the use of a self-reported HRQoL outcome from a large multicentre RCT comparing LMI to HI exercise, with limited loss to follow-up. The exercise intervention followed a thoroughly standardised protocol, ensuring that the intervention was carried out in a very rigorous manner in which both the exercise delivered, and the exercise volume and intensity were monitored.

The main limitation of our study was that, for comparison, we used a smaller non-randomised UC group included before the RCT, making the groups not directly comparable. However, the main aim of the Phys-Can was to compare different levels of exercise intensity; thus, we considered it unethical to design an RCT randomising participants to usual care since there are strong evidence that exercise have a positive effect on fatigue and HRQoL [8]. In the present study, the measured characteristics of the participants in UC were almost as similar as in the RCT, except that a smaller proportion in the UC group received chemotherapy, and analyses were adjusted accordingly. To try to avoid bias, we used a mixed model approach with adjustment for auxiliary variables to handle missing data. Another limitation was that our results were based on exploratory analysis with multiple endpoints of the subscales of HRQoL, and the statistical power analysis was based on the main outcome physical fatigue in the RCT. Thus, the internal validity of our findings may be limited. Moreover, our results may not be generalisable to all patients with breast, colorectal and prostate cancer receiving (neo)adjuvant oncological treatment. Those who consented to participation were a relatively healthy group compared with the general cancer population. Only 29% of the eligible patients chose to participate in the RCT, and this sample might have been biased, as they were probably motivated to exercise. Also, participants with breast cancer were over-represented.

In conclusion, patients with breast, colorectal and prostate cancer can exercise at either intensity level according to their personal preferences and will still benefit from short-term and long-term improvements in HRQoL. The key clinical message is to recommend patients to exercise according to their personal condition. Exercise on low-to-moderate intensity is sufficient to achieve beneficial health effects and to improve symptoms of the oncological treatment. Future studies are needed to confirm our results in broader clinical populations.

## Supplementary Information

Below is the link to the electronic supplementary material.

Supplementary file1 (DOCX 80 KB)

## Data Availability

The data that support the findings of this study are available from the corresponding author upon request.
