# Supplementary material for: Short- and long-term effect of high versus low-to-moderate intensity exercise to optimise health-related quality of life after oncological treatment—results from the Phys-Can project
Source: Support Care Cancer. 2022 Apr 7;30(7):5949–63. doi: 10.1007/s00520-022-07016-3 (PMC9135802; doi:10.1007/s00520-022-07016-3)
Supplement: Supplementary file 1 — Supplementary file1 (DOCX 80 KB) [file 520_2022_7016_MOESM1_ESM.docx]

| Table S1: Longitudinal crude mean scores of EORTC QLQ-C30 and EORTC QLQ-BR23 of high- and low-moderate training intensity and usual care in patients with breast cancer. | | | | | |
| --- | --- | --- | --- | --- | --- |
| *Mean (SD)* | | Baseline | 3 months | 6 months | 18 months |
| EORTC QLQ-C30 | | | | | |
| Maximum number of respondents | HI | 220 | 165 | 169 | 162 |
|  | LMI | 226 | 156 | 172 | 164 |
|  | UC | 74 | 47 | 45 | 39 |
| Global health status^1^ | HI | 65 (20) | 62 (22) | 73 (19) | 73 (19) |
|  | LMI | 63 (20) | 62 (22) | 72 (18) | 73 (17) |
|  | UC | 68 (19) | 62 (21) | 65.9 (21) | 74 (17) |
| Functioning^1^ | | | | | |
| Physical functioning | HI | 89 (14) | 83 (17) | 89 (14) | 89 (14) |
|  | LMI | 88 (13) | 85 (14) | 90 (11) | 90 (12) |
|  | UC | 89 (12) | 87 (11) | 88 (13) | 91 (12) |
| Role functioning | HI | 71 (30) | 66 (32) | 81 (25) | 85 (24) |
|  | LMI | 70 (29) | 68 (30) | 83 (21) | 85 (23) |
|  | UC | 74 (28) | 71 (31) | 79 (24) | 99 (19) |
| Emotional functioning | HI | 68 (21) | 75 (21) | 76 (23) | 79 (21) |
|  | LMI | 67 (22) | 76 (22) | 77 (19) | 80 (17) |
|  | UC | 74 (22) | 75 (23) | 81 (21) | 77 (19) |
| Cognitive functioning | HI | 81 (22) | 76 (24) | 82 (22) | 85 (19) |
|  | LMI | 80 (22) | 75 (24) | 79 (19) | 83 (17) |
|  | UC | 82 (23) | 81 (21) | 82 (20) | 84 (20) |
| Social functioning | HI | 79 (22) | 72 (26) | 81 (23) | 84 (22) |
|  | LMI | 76 (23) | 71 (26) | 83 (21) | 86 (18) |
|  | UC | 82 (21) | 76 (26) | 82 (24) | 86 (22) |
| Symptoms^2^ | | | | | |
| Fatigue | HI | 29 (20) | 41 (25) | 26 (20) | 25 (21) |
|  | LMI | 32 (23) | 40 (25) | 27 (18) | 25 (18) |
|  | UC | 25 (17) | 36 (23) | 31 (23) | 25 (20) |
| Nausea and vomiting | HI | 3.9 (9.9) | 8.2 (14) | 2.6 (8.1) | 2.2 (6.5) |
|  | LMI | 3.2 (8.1) | 7.6 (13) | 2.8 (8.7) | 2.6 (9.0) |
|  | UC | 2.9 (7.0) | 6.4 (12) | 1.9 (8.1) | 2.6 (12) |
| Pain | HI | 20 (23) | 27 (28) | 22 (25) | 21 (23) |
|  | LMI | 24 (24) | 25 (26) | 18 (20) | 20 (24) |
|  | UC | 18 (20) | 16 (23) | 17 (23) | 19 (25) |
| Dyspnea | HI | 9.8 (19) | 23 (29) | 9.1 (20) | 9.2 (20) |
|  | LMI | 9.2 (18) | 21 (27) | 11 (19) | 8.2 (18) |
|  | UC | 5.0 (14) | 11 (19) | 10 (20) | 9.4 (22) |
| Insomnia | HI | 34 (30) | 38 (31) | 34 (31) | 29 (30) |
|  | LMI | 35 (31) | 37 (33) | 33 (30) | 29 (27) |
|  | UC | 31 (31) | 36 (32) | 33 (32) | 33 (31) |
| Appetite loss | HI | 10 (20) | 14 (23) | 5.3 (15) | 6.2 (18) |
|  | LMI | 11 (19) | 15 (26) | 5.1 (13) | 4.1 (16) |
|  | UC | 6.3 (9) | 9.9 (21) | 4.4 (17) | 5.1 (20) |
| Constipation | HI | 5.6 (15) | 13 (23) | 6.5 (16) | 6.4 (16) |
|  | LMI | 6.1 (15) | 14 (24) | 10 (21) | 9.2 (19) |
|  | UC | 5.9 (14) | 11 (24) | 7.4 (17) | 10 (21) |
| Diarrhea | HI | 8.1 (19) | 13 (24) | 6.3 (17) | 5.6 (16) |
|  | LMI | 8.5 (19) | 16 (24) | 8.1 (19) | 6.1 (17) |
|  | UC | 8.6 (20) | 14 (22) | 10. (23) | 7.7 (22) |
| Financial difficulties | HI | 12 (23) | 11 (21) | 11 (22) | 7.8 (18) |
|  | LMI | 13 (25) | 13 (24) | 11 (20) | 6.1 (17) |
|  | UC | 11 (22) | 9.4 (22) | 8.9 (24) | 4.3 (14) |
| EORTC QLQ-BR 23 | | | | | |
| Maximum number of respondents | HI | 214 | 162 | 167 | 161 |
|  | LMI | 226 | 159 | 171 | 162 |
|  | UC | 72 | 48 | 46 | 39 |
| Body image^1^ | HI | 80 (23) | 71 (27) | 76 (25) | 80 (23) |
|  | LMI | 78 (27) | 68 (29) | 72 (25) | 79 (24) |
|  | UC | 83 (21) | 79 (22) | 80 (24) | 81 (23) |
| Sexual functioning^2^ | HI | 20 (22) | 16 (20) | 22 (22) | 24 (23) |
|  | LMI | 22 (21) | 17 (20) | 21 (22) | 25 (24) |
|  | UC | 22 (23) | 25 (20) | 26 (19) | 30 (23) |
| Sexual enjoyment^2^ | HI | 65 (25) | 58 (27) | 58 (30) | 64 (28) |
|  | LMI | 61 (27) | 52 (28) | 54 (25) | 59 (29) |
|  | UC | 66 (26) | 52 (25) | 57 (20) | 65 (26) |
| Future perspective^1^ | HI | 50 (29) | 57 (33) | 58 (32) | 62 (31) |
|  | LMI | 49 (30) | 56 (30) | 56 (28) | 62 (27) |
|  | UC | 60 (26) | 65 (26) | 61 (28) | 67 (29) |
| Systemic therapy^2^ | HI | 14 (13) | 34 (22) | 21 (16) | 17 (14) |
|  | LMI | 14 (14) | 35 (22) | 22 (14) | 17 (13) |
|  | UC | 12 (10) | 26 (17) | 19 (15) | 19 (18) |
| Breast symptoms^2^ | HI | 27 (18) | 15 (17) | 18 (19) | 13 (15) |
|  | LMI | 29 (22) | 14 (14) | 18 (18) | 12 (13) |
|  | UC | 22 (18) | 16 (16) | 21 (20) | 13 (18) |
| Arm symptoms^2^ | HI | 13 (17) | 10 (16) | 11 (15) | 10 (16) |
|  | LMI | 15 (17) | 9.4 (14) | 12 (17) | 11 (15) |
|  | UC | 14 (13) | 10 (17) | 11 (17) | 8.5 (21) |
| Hair loss^2^ | HI | 2.9 (13) | 23 (34) | 7.7 (23) | 5.9 (18) |
|  | LMI | 3.3 (16) | 26 (37) | 11 (28) | 5.9 (20) |
|  | UC | 2.4 (13) | 14 (26) | 6.1 (23) | 3.6 (17) |
| Note: Results are presented as means ± S.D. (range). HI: high intensity exercise; LMI: low-to-moderate intensity exercise. UC: usual care. Results based on all available data. Missing data due to drop-outs in the study and lost to follow up. 1. Higher score indicate better HRQoL = better functioning. 2.Higher score indicate worse HRQoL = more symptoms/problems. | | | | | |

| Table S2: Longitudinal crude mean scores of EORTC QLQ-C30 and EORTC QLQ-CR29 of high- and low-moderate training intensity and usual care in patients with colorectal cancer. | | | | | |
| --- | --- | --- | --- | --- | --- |
| *Mean (SD)* | | Baseline | 3 months | 6 months | 18 months |
| EORTC QLQ-C30 | | | | | |
| Maximum number of respondents | HI | 11 | 7 | 9 | 8 |
|  | LMI | 11 | 10 | 11 | 10 |
|  | UC | 4 | 2 | 3 | 3 |
| Global health status^1^ | HI | 71 (20) | 76 (18) | 83 (14) | 69 (28) |
|  | LMI | 62 (28) | 66 (25) | 75 (22) | 73 (24) |
|  | UC | 69 (16) | 75 (12) | 61 (32) | 75 (22) |
| Functioning^1^ | | | | | |
| Physical functioning | HI | 83.0 (10.5) | 93 (7.7) | 96 (3.5) | 83 (16) |
|  | LMI | 89.7 (9.1) | 83 (17) | 90 (12) | 85 (16) |
|  | UC | 90.0 (11.5) | 97 (4.7) | 73 (35) | 82 (31) |
| Role functioning | HI | 63.6 (38.6) | 91 (19) | 93 (15) | 75 (30) |
|  | LMI | 60.6 (38.2) | 73 (27) | 88 (15) | 80 (23) |
|  | UC | 87.5 (25.0) | 67 (47) | 61 (42) | 83 (29) |
| Emotional functioning | HI | 74 (18.7) | 83 (17) | 88 (10) | 82 (21) |
|  | LMI | 75.8 (10.8) | 84 (13) | 86 (18) | 91 (8.3) |
|  | UC | 83.3 (11.8) | 92 (12) | 83 (29) | 89 (19) |
| Cognitive functioning | HI | 83.3 (16.7) | 74 (9) | 82 (18) | 83 (20) |
|  | LMI | 87.9 (10.8) | 78 (19) | 86 (16) | 93 (8.6) |
|  | UC | 95.8 (8.3) | 92 (12) | 89 (19) | 94 (9.6) |
| Social functioning | HI | 77.3 (18.7) | 91 (19) | 89 (14) | 77 (20) |
|  | LMI | 75.8 (21.6) | 77 (18) | 85 (20) | 93 (12) |
|  | UC | 83.3 (13.6) | 75 (35) | 78 (39) | 94 (9.6) |
| Symptoms^2^ | | | | | |
| Fatigue | HI | 32 (17) | 25 (19) | 15 (13) | 31 (24) |
|  | LMI | 30 (20) | 39 (21) | 21 (15) | 23 (22) |
|  | UC | 31 (19) | 22 (31) | 44 (51) | 22 (29) |
| Nausea and vomiting | HI | 4.5 (7.8) | 2.4 (6.3) | 11 (22) | 4.2 (12) |
|  | LMI | 6.1 (14) | 8.3 (14) | 0 (0) | 1.7 (5.3) |
|  | UC | 4.2 (8.3) | 17 (24) | 0 (0) | 33 (58) |
| Pain | HI | 20 (18) | 7.1 (8.9) | 13 (18) | 13 (15) |
|  | LMI | 17 (26) | 17 (30) | 21 (32) | 30 (34) |
|  | UC | 8.3 (17) | 8.3 (12) | 28 (9.6) | 0 (0) |
| Dyspnea | HI | 3.0 (10) | 0 (0) | 0 (0) | 21 (17) |
|  | LMI | 3.3 (11) | 30 (37) | 9.1 (16) | 13 (28) |
|  | UC | 0 (0) | 17 (24) | 44 (51) | 11 (19) |
| Insomnia | HI | 27 (36) | 38 (36) | 33 (33) | 38 (38) |
|  | LMI | 30 (18) | 17 (18) | 18 (27) | 30 (33) |
|  | UC | 17 (19) | 33 (0) | 33 (33) | 33 (0) |
| Appetite loss | HI | 18 (17) | 0 (0) | 7.4 (15) | 17 (25) |
|  | LMI | 12 (23) | 10 (23) | 0 (0) | 6.7 (14) |
|  | UC | 8.3 (17) | 17 (24) | 22 (39) | 33.3 (58) |
| Constipation | HI | 6.1 (14) | 9.5 (16) | 11 (24) | 8.3 (15) |
|  | LMI | 21 (34) | 17 (24) | 15 (23) | 17 (32) |
|  | UC | 17 (33) | 0 (0) | 0 (0) | 33.3 (58) |
| Diarrhea | HI | 15 (17) | 0 (0) | 0 (0) | 21 (35) |
|  | LMI | 18 (23) | 20 (32) | 6.1 (14) | 6.7 (14) |
|  | UC | 0 (0) | 33 (0) | 22 (19) | 33 (33) |
| Financial difficulties | HI | 6.1 (20) | 14 (38) | 11 (33) | 21 (40) |
|  | LMI | 15 (27) | 20 (28) | 15 (23) | 13 (23) |
|  | UC | 0 (0) | 0 (0) | 0 (0) | 0 (0) |
| EORTC QLQ-CR29 | | | | | |
| Maximum number of respondents | HI | 11 | 7 | 9 | 8 |
|  | LMI | 11 | 9 | 10 | 10 |
|  | UC | 4 | 1 | 3 | 3 |
| Body image^1^ | HI | 86 (19) | 79 (18) | 85 (16) | 75 (24) |
|  | LMI | 86 (25) | 96 (7.9) | 83 (33) | 83 (32) |
|  | UC | 94 (11) | 78 (0) | 82 (32) | 96 (6.4) |
| Anxiety^2^ | HI | 52 (27) | 62 (36) | 70 (31) | 67 (27) |
|  | LMI | 64 (23) | 70 (20) | 73 (34) | 73 (21) |
|  | UC | 67 (27) | 100 (0) | 78 (39) | 89 (19) |
| Weigth^2^ | HI | 91 (16) | 95 (13) | 85 (18) | 86 (18) |
|  | LMI | 91 (16) | 85 (18) | 83 (32) | 80 (32) |
|  | UC | 75 (17) | 100 (0) | 89 (19) | 78 (39) |
| Sexual interest (men)^1^ | HI | 33 (25) | 33 (30) | 48 (26) | 33 (39) |
|  | LMI | 28 (14) | 25 (17) | 27 (15) | 40 (28) |
|  | UC | 44 (19) | 33 (0) | 44 (19) | 44 (19) |
| Sexual interest (women)^1^ | HI | 0 (0) | 0 (0) | 16.7 (23.6) | 0 (0) |
|  | LMI | 6.7 (15) | 7 (15) | 6.7 (15) | 13 (18) |
|  | UC | 33 (0) | - | 0 (0) | 0 (0) |
| Urinary frequency^2^ | HI | 41 (26) | 41 (29) | 43 (25) | 33 (24) |
|  | LMI | 27 (18) | 30 (22) | 23 (21) | 13 (21) |
|  | UC | 29 (16) | 67 (0) | 33 (0) | 28 (9.6) |
| Blood and mucus in stool^2^ | HI | 3.0 (6.7) | 0 (0) | 0 (0) | 2.1 (5.9) |
|  | LMI | 4.5 (7.8) | 3.7 (11) | 1.7 (5.3) | 0 (0) |
|  | UC | 0 (0) | 0 (0) | 0 (0) | 0 (0) |
| Stool frequency^2^ | HI | 15 (19) | 9.5 (16) | 3.7 (11) | 25 (39) |
|  | LMI | 6.1 (11) | 5.6 (8.3) | 1.7 (5.3) | 5.0 (11) |
|  | UC | 0 (0) | 0 (0) | 5.6 (9.6) | 0 (0) |
| Urinary incontinence^2^ | HI | 3.0 (10) | 14 (18) | 11 (17) | 13 (17) |
|  | LMI | 0 (0) | 0 (0) | 0 (0) | 3.3 (11) |
|  | UC | 0 (0) | 0 (0) | 0 (0) | 0 (0) |
| Dysuria^2^ | HI | 18 (35) | 4.8 (13) | 7.4 (15) | 0 (0) |
|  | LMI | 0 (0) | 0 (0) | 0 (0) | 3.3 (11) |
|  | UC | 0 (0) | 0 (0) | 0 (0) | 0 (0) |
| Abdominal pain^2^ | HI | 18 (27) | 4.8 (13) | 7.4 (15) | 13 (17) |
|  | LMI | 10 (16) | 3.7 (11) | 7.4 (15) | 6.7 (14) |
|  | UC | 17 (19) | 33 (0) | 22 (19) | 0 (0) |
| Buttock pain^2^ | HI | 12 (23) | 9.5 (16) | 7.4 (15) | 4.2 (12) |
|  | LMI | 18 (27) | 11 (17) | 6.7 (14) | 10 (16) |
|  | UC | 8.3 (17) | 0 (0) | 0 (0) | 0 (0) |
| Bloating^2^ | HI | 12 (17) | 4.8 (13) | 11 (17) | 17 (18) |
|  | LMI | 13 (17) | 11 (17) | 15 (24) | 13 (23) |
|  | UC | 17 (19) | 33 (0) | 11 (19) | 22 (19) |
| Dry mouth^2^ | HI | 18 (27) | 38 (41) | 30 (39) | 29 (41) |
|  | LMI | 24 (21) | 41 (22) | 20 (17) | 17 (18) |
|  | UC | 16 (19) | 33 (0) | 11 (19) | 0 (0) |
| Hair loss^2^ | HI | 9.1 (22) | 0 (0) | 3.7 (11) | 29 (49) |
|  | LMI | 6.1 (14) | 33 (31) | 10 (16) | 6.7 (14) |
|  | UC | 0 (0) | 0 (0) | 33 (58) | 11 (19) |
| Taste^2^ | HI | 6.1 (20) | 0 (0) | 15 (24) | 9.5 (16) |
|  | LMI | 3.0 (10) | 19 (24) | 3.3 (11) | 0 (0) |
|  | UC | 0 (0) | 0 (0) | 22 (39) | 0 (0) |
| Flatulence^2^ | HI | 24 (22) | 24 (25) | 26 (36) | 33 (31) |
|  | LMI | 15 (17) | 19 (18) | 13 (23) | 10 (23) |
|  | UC | 8.3 (17) | 0 (0) | 11 (19) | 22 (19) |
| Fecal incontinence^2^ | HI | 0 (0) | 4.8 (13) | 7.4 (15) | 13 (25) |
|  | LMI | 0 (0) | 0 (0) | 3.3 (11) | 3.3 (11) |
|  | UC | 0 (0) | 0 (0) | 0 (0) | 0 (0) |
| Sore skin^2^ | HI | 12 (23) | 4.8 (13) | 3.7 (11) | 8.3 (15) |
|  | LMI | 15 (23) | 15 (18) | 6.7 (14) | 6.7 (14) |
|  | UC | 0 (0) | 0 (0) | 22 (19) | 0 (0) |
| Embarrassment^2^ | HI | 25 (17) | 33 (27) | 33 (39) | 22 (19) |
|  | LMI | 67 (47) | 0 (0) | 0 (0) | 0 (0) |
|  | UC | 0 (0) | 0 (0) | 0 (0) | 0 (0) |
| Stoma care problems^2^ | HI | 0 (0) | 8.3 (17) | 17 (19) | 11 (19) |
|  | LMI | 17 (24) | 33 (0) | 0 (0) | 0 (0) |
|  | UC | 0 (0) | 0 (0) | 0 (0) | 0 (0) |
| Impotence (men^)1^ | HI | 58 (35) | 53 (18) | 50 (18) | 62 (41) |
|  | LMI | 39 (33) | 25 (32) | 53 (51) | 27 (28) |
|  | UC | 22 (39) | 67 (0) | 33 (33) | 44 (19) |
| Dyspareunia (women)^1^ | HI | 0 (0) | 0 (0) | 67 (47) | 100 (0) |
|  | LMI | 0 (0) | 0 (0) | 0 (0) | 8.3 (17) |
|  | UC | 33 (0) | - | 0 (0) | 0 (0) |
| Note: Results are presented as means ± S.D. (range). HI: high intensity exercise; LMI: low-to-moderate intensity exercise. UC: usual care. Results based on all available data. Missing data due to drop-outs in the study and lost to follow up. 1. Higher score indicate better HRQoL = better functioning. 2.Higher score indicate worse HRQoL = more symptoms/problems. | | | | | |

| Table S3: Longitudinal crude mean scores of EORTC QLQ-C30 and EORTC QLQ-PR25 of high- and low-moderate training intensity and usual care in patients with prostate cancer. | | | | | |
| --- | --- | --- | --- | --- | --- |
| *Mean (SD)* | | Baseline | 3 months | 6 months | 18 months |
| EORTC QLQ-C30 | | | | | |
| maximum number of respondents | HI | 48 | 38 | 42 | 38 |
|  | LMI | 46 | 40 | 46 | 40 |
|  | UC | 10 | 7 | 8 | 7 |
| Global health status^1^ | HI | 79 (19) | 75 (21) | 82 (16) | 80 (18) |
|  | LMI | 75 (19) | 76 (20) | 78 (18) | 77 (21) |
|  | UC | 81 (15) | 73 (20) | 79 (17) | 69 (24) |
| Functioning^1^ | | | | | |
| Physical functioning | HI | 90 (12) | 90 (12) | 91 (12) | 88 (15) |
|  | LMI | 91 (17) | 89 (18) | 90 (16) | 87 (19) |
|  | UC | 95 (7.6) | 91 (12) | 92 (11) | 91 (11) |
| Role functioning | HI | 94 (12) | 91 (21) | 91 (21) | 87 (24) |
|  | LMI | 94 (17) | 95 (15) | 95 (15) | 88 (26) |
|  | UC | 95 (11) | 92 (13) | 92 (13) | 86 (15) |
| Emotional functioning | HI | 89 (14) | 85 (18) | 89 (14) | 89 (15) |
|  | LMI | 87 (13) | 86 (15) | 89 (11) | 89 (14) |
|  | UC | 83 (14) | 89 (19) | 92 (15) | 88 (14) |
| Cognitive functioning | HI | 90 (13) | 86 (18) | 87 (16.4) | 86 (12) |
|  | LMI | 88 (15) | 89 (16) | 88 (17.4) | 85 (18) |
|  | UC | 93 (8.6) | 95 (8.1) | 91 (8.9) | 93 (8.9) |
| Social functioning | HI | 90 (19) | 88 (15) | 88 (14) | 89 (18) |
|  | LMI | 88 (17) | 82 (23) | 87 (19) | 81 (27) |
|  | UC | 85 (21) | 86 (15) | 88 (16) | 88 (16) |
| Symptoms^2^ | | | | | |
| Fatigue | HI | 18 (18) | 25 (19) | 21.4 (20.3) | 22.8 (22.2) |
|  | LMI | 21 (21) | 22 (20) | 19.4 (20.0) | 24.6 (24.6) |
|  | UC | 10 (11) | 24 (21) | 27.8 (25.2) | 27.8 (30.3) |
| Nausea and vomiting | HI | 1.0 (5.3) | 3.5 (7.9) | 2.4 (8.7) | 0.5 (2.7) |
|  | LMI | 2.2 (6.7) | 1.7 (5.1) | 1.1 (4.2) | 2.5 (6.0) |
|  | UC | 3.3 (11) | 4.8 (13) | 2.1 (5.9) | 0 (0) |
| Pain | HI | 14 (17) | 17 (20) | 13 (20) | 23 (27) |
|  | LMI | 13 (22) | 15 (24) | 9.4 (17) | 12 (18) |
|  | UC | 15 (18) | 21 (23) | 17 (24) | 29 (28) |
| Dyspnea | HI | 13 (21) | 12 (21) | 7.9 (18) | 12 (21) |
|  | LMI | 8.1 (15) | 9.2 (17) | 6.5 (17) | 13 (27) |
|  | UC | 6.7 (14) | 19 (38) | 17 (25) | 19 (38) |
| Insomnia | HI | 12 (19) | 26 (26) | 17 (20) | 19 (20) |
|  | LMI | 14 (25) | 19 (25) | 21 (27) | 18 (31) |
|  | UC | 17 (18) | 9.5 (16) | 17 (25) | 19 (38) |
| Appetite loss | HI | 3.5 (16) | 6.1 (17) | 1.6 (7.2) | 4.5 (16) |
|  | LMI | 3.6 (16) | 3.4 (17) | 2.2 (11) | 6.8 (17) |
|  | UC | 6.7 (21) | 4.8 (13) | 0 (0) | 0 (0) |
| Constipation | HI | 4.9 (14) | 12.3 (23) | 4.9 (14) | 7.2 (16) |
|  | LMI | 6.5 (15) | 5.8 (13) | 4.4 (14) | 5.8 (15) |
|  | UC | 6.7 (21) | 4.8 (13) | 13 (25) | 0 (0) |
| Diarrhea | HI | 8.3 (20) | 16.2 (24) | 12 (21) | 9.9 (17) |
|  | LMI | 5.2 (14) | 9.2 (19) | 14 (21) | 14 (25) |
|  | UC | 3.3 (11) | 14.3 (18) | 9.5 (16) | 4.8 (13) |
| Financial difficulties | HI | 1.4 (6.7) | 6.1 (19) | 4.8 (19) | 2.7 (9.2) |
|  | LMI | 4.3 (15) | 0.8 (5.3) | 2.9 (12) | 3.3 (10) |
|  | UC | 3.3 (11) | 4.8 (13) | 4.8 (13) | 0 (0) |
| EORTC QLQ-PR25 | | | | | |
| Maximum number of respondents | HI | 48 | 38 | 41 | 38 |
|  | LMI | 44 | 40 | 46 | 41 |
|  | UC | 10 | 7 | 8 | 7 |
| Sexual activity^1^ | HI | 31 (22) | 21 (22) | 22 (25) | 22 (27) |
|  | LMI | 38 (26) | 23 (22) | 26 (24) | 27 (23) |
|  | UC | 50 (27) | 22 (27) | 35 (27) | 41 (29) |
| Sexual functioning^1^ | HI | 65 (14) | 59 (20) | 51 (19) | 52 (13) |
|  | LMI | 66 (17) | 63 (14) | 60 (17) | 57 (17) |
|  | UC | 69 (9.7) | 33 (12) | 53 (25) | 63 (13) |
| Urinary symptoms^2^ | HI | 18 (15) | 30 (17) | 25 (16) | 27 (19) |
|  | LMI | 18 (15) | 29 (19) | 26 (17) | 25 (17) |
|  | UC | 20 (22) | 41 (21) | 25 (21) | 24 (23) |
| Bowel symptoms^2^ | HI | 1.6 (4.5) | 6.9 (11) | 6.7 (9.0) | 8.9 (8.2) |
|  | LMI | 4.1 (7.5) | 6.3 (9.6) | 8.1 (10) | 7.2 (10) |
|  | UC | 3.3 (5.8) | 3.6 (4.5) | 6.0 (9.3) | 6.0 (9.3) |
| Hormonal treatment symptoms^2^ | HI | 7.4 (7.5) | 15 (9.0) | 16 (9.9) | 20 (13) |
|  | LMI | 11 (13) | 16 (12) | 16 (11) | 19 (14) |
|  | UC | 7.4 (6.1) | 21 (12) | 26 (16) | 16 (12) |
| Incontinence aid^2^ | HI | 22 (40) | 22 (27) | 17 (36) | 29 (30) |
|  | LMI | 17 (18) | 0 (0) | 11 (17) | 20 (28) |
|  | UC | 0 (0) | 33 (0) | 0 (0) | 0 (0) |
| Note: Results are presented as means ± S.D. (range). HI: high intensity exercise; LMI: low-to-moderate intensity exercise. UC: usual care. Results based on all available data. Missing data due to drop-outs in the study and lost to follow up. 1. Higher score indicate better HRQoL = better functioning. 2.Higher score indicate worse HRQoL = more symptoms/problems. | | | | | |
